# Mental health first aid training for Australian medical and nursing students: an evaluation study

**DOI:** 10.1186/s40359-015-0069-0

**Published:** 2015-04-12

**Authors:** Kathy S Bond, Anthony F Jorm, Betty A Kitchener, Nicola J Reavley

**Affiliations:** Mental Health First Aid Australia, Level 6/369 Royal Parade, Parkville, VIC 3052 Australia; Centre for Mental Health, Melbourne School of Population and Global Health, The University of Melbourne, Level 4/207 Bouverie St., Parkville, VIC 3010 Australia; School of Psychology, Deakin University, Geelong, Victoria Australia

**Keywords:** Nursing students, Medical students, Mental health first aid training, Evaluation

## Abstract

**Background:**

The role and demands of studying nursing and medicine involve specific stressors that may contribute to an increased risk for mental health problems. Stigma is a barrier to help-seeking for mental health problems in nursing and medical students, making these students vulnerable to negative outcomes including higher failure rates and discontinuation of study. Mental Health First Aid (MHFA) is a potential intervention to increase the likelihood that medical and nursing students will support their peers to seek help for mental health problems. This study aimed to evaluate the effectiveness of a tailored MHFA course for nursing and medical students.

**Methods:**

Nursing and medical students self-selected into either a face-to-face or online tailored MHFA course. Four hundred and thirty-four nursing and medical students completed pre- and post-course surveys measuring mental health first aid intentions, mental health literacy, confidence in providing help, stigmatising attitudes and satisfaction with the course.

**Results:**

The results of the study showed that both the online and face-to-face courses improved the quality of first aid intentions towards a person experiencing depression, and increased mental health literacy and confidence in providing help. The training also decreased stigmatizing attitudes and desire for social distance from a person with depression.

**Conclusion:**

Both online and face-to-face tailored MHFA courses have the potential to improve outcomes for students with mental health problems, and may benefit the students in their future professional careers.

## Background

Evidence from a national survey suggests that Australian tertiary students have a higher rate of moderate psychological distress compared to non-students of the same age (Cvetkovski et al. 2012). Moreover, the role and demands of studying nursing and medicine involve specific stressors that may further increase this distress as students progress through their courses. A number of studies have investigated mental health problems in nursing and medical students. For example, a cross-sectional study of 431 Australian undergraduate nursing students found burnout and stress levels increased across their years of study. By the completion of their course, up to 20% of students were reporting signs of serious maladaptive fatigue or stress (Rella et al. 2009). A systematic literature review identified two main sources of stress in nursing students: academic factors (e.g. workload and problems associated with studying) and clinical factors (e.g. fear of unknown situations, mistakes with patients or handling of technical equipment) (Pulido Martos et al. 2012). A study of Australian nursing students across the three years of their nursing program identified difficulty with studies and finances as the main stressors (Lo 2002).

The mental health of medical students and doctors in Australia is an ongoing concern within the medical profession and community (Elliot et al. 2010; Schlicht et al. 1990). While medical students have similar psychological wellbeing to the general student population before embarking on their studies (Rossal et al. 1997; Carson et al. 2000; Singh et al. 2004), international and Australian research suggests that their psychological wellbeing declines as they progress through their study (Aktekin et al. 2001; Henning et al. 1998; Toews et al. 1993; Dahlin et al. 2010; Psujek et al. 2004; Biro et al. 2010; Dyrbye et al. 2006; Miller and Chung 2009; Willcock et al. 2004). One study that measured psychiatric morbidity for common mental illnesses in Australian medical students found a significant increase in scores from enrollment to the end of their internship, with final measure scores increasing past the cutoff for potential psychiatric morbidity (Willcock et al. 2004). The international literature supports this finding, noting a number of factors that are significant stressors for medical students including volume of workload; worry about academic performance; high-stake examinations; bullying from fellow clinical staff, students, residents and interns; supervisors who are stressed, depressed or burned out (Feudtner et al. 1994; Richman et al. 1992; Kassebaum and Cutler 1998; Wear 2002); and exposure to human suffering (Wolf et al. 1988; Supe 1998; Guthrie et al. 1995; Vitaliano et al. 1984).

A number of negative outcomes can result from mental health problems in tertiary students, including delay or discontinuation of university studies, absenteeism, reduced productivity, and higher failure rates (Andrews and Wilding 2004; Arria et al. 2013; James et al. 2010). Early intervention for mental health problems in nursing and medical students is important to reducing these and other negative outcomes. However, stigma may be a barrier to help-seeking. In a survey of Australian and New Zealand medical students, 55% agreed there was a stigma attached to being a medical student with psychological distress and 72% agreed there was a stigma attached to being a medical student with a diagnosed mental illness (Elliot and Tan 2010). In a 2011 study of Australian medical students, 20% felt they needed to conceal mental and emotional problems (Walter et al. 2013).

Stigma and associated barriers to help-seeking are also present after graduation. Documented barriers for medical practitioners seeking help for a mental health problem include (Elliot and Tan 2010):

- Concerns over lack of confidentiality
- Embarrassment and fear of being perceived as weak
- Perceived impact on career development
- Perceived impact on peers and patients
- Expectation that they should work while unwell
- Perception that it reflects on their professional integrity, e.g. requirement for mandatory reporting
- Stigma of health professionals themselves having illness.

Similarly, nurses who have mental health problems (Joyce et al. 2012) have reported that they often experienced a lack of acceptance from their colleagues, e.g. being gossiped about and denigrated in front of other nurses. Many nurses avoid disclosing their mental health problems because of their own stigmatisation of other colleagues.

Young people with mental health problems often prefer to seek assistance from friends and family (Jorm et al. 2007; Reavley et al. 2012a). Nursing students identify their friends as a major source of support, especially during high stress situations like clinical placements (Chapman and Orb 2001). A 2009 survey of Australian medical students found that 88% expressed a preference for seeking help from a friend if they are depressed (Rong et al. 2009). However, another survey of medical students at the same university found that 19% felt not at all supported mentally or emotionally, while 36% felt only a little supported (Walter et al. 2013). This may indicate that while medical students find friends helpful in managing stress, they may not know how to provide specific support to a friend experiencing mental health problems.

A potential intervention which may increase mental health knowledge in nursing and medical students and increase the likelihood that they may offer support to a peer who is experiencing mental health problems is mental health first aid training. Mental Health First Aid (MHFA) is a 12-hour training course which teaches members of the public how to respond to a person who is developing a mental illness or experiencing a mental health crisis (Jorm and Kitchener 2011). The course has been extensively evaluated (Hadlaczky et al. 2014), including five controlled trials. These studies have shown that course participants have increased mental health first aid knowledge, improved attitudes to appropriate mental health treatments, decreased stigma towards those with mental health problems, and increased confidence in providing support to people experiencing mental health problems. The course has also been shown to be similarly effective in specific populations, including high school teachers (Jorm et al. 2010a), financial counsellors (Bond, K, Jorm, A, Kitchener, B and Reavley, N. Submitted), and pharmacy students (O'Reilly et al. 2011). In 2013 the Australian Government Department of Health provided funding for the adaptation and provision of the 12-hour mental health first aid course in two modes – face-to-face and on-line. The Mental Health First Aid Australia Standard MHFA course (for adults providing MHFA to adults) was tailored to meet the needs of nursing and medical students to better support their peers. Both face-to-face and on-line versions were offered to allow students a choice of mode depending on their preference and time schedules.

Online learning has the potential to provide flexible access to learning resources for those who are unable to attend face-to-face learning. Its other benefits include a more cost-efficient delivery of course material and increased numbers of students being able to access the material (Means et al. 2009). In spite of these benefits, online learning must produce equal or better educational outcomes in order to be deemed beneficial to learners and educational institutions. Two meta-analyses of online versus face-to-face learning were conducted in 2006 and 2009, and found online learning to be slightly more beneficial than face-to-face learning (Means et al. 2009; Sitzmann et al. 2006). However, the authors noted methodological issues that made it difficult to determine if delivery method, student time spent on the material, curriculum or pedagogy produced the results. They concluded that online and face-to-face delivery methods are comparable. A literature review of 76 studies from the medical, nursing and dental literature on the effectiveness of online learning found similar results to the meta-analyses (Chumley-Jones et al. 2002).

The aims of this project were to investigate the impact of MHFA training for nursing and medical students on (1) mental health first aid intentions, (2) mental health literacy, (3) confidence in providing help, (4) stigmatising attitudes and (5) satisfaction with the course. The project also aimed to compare the outcomes of on-line and face-to-face versions of the course. Participants were asked to complete a pre-course questionnaire, participate in the MHFA course, and complete a post-course questionnaire.

## Methods

### Description of the tailored MHFA course

The tailored standard MHFA (Kitchener et al. 2013a) course (for adults providing MHFA to adults) includes some aspects of the Youth MHFA (Kelly et al. 2013) course (for adults providing MHFA to adolescents), specifically a section on eating disorders. This was done because many nursing and medical students are in the 16–24 age range, which is a typical age for onset of eating disorders (Oakley Browne et al. 2006). In addition, supplementary booklets and new videos, with examples of how to provide mental health first aid to fellow students, were developed (Bovopoulos et al. 2013; Kitchener et al. 2013b).

All students who enrolled in the tailored course received a copy of the Mental Health First Aid Manual, 3^rd^ ed. (Kitchener et al. 2013a) and the relevant supplementary manual (Bovopoulos et al. 2013; Kitchener et al. 2013b). The course was delivered as either a 13-hour face-to-face course or an online course to allow the students to choose the method of delivery that best fit their schedule and preference for learning, thus increasing the number of student to receive the training. When a student enrolled in the online course they were provided with an account so they could log-in and complete the course. Online students who did not complete the course were emailed reminders every 2–3 months over the first year of the funding (1 July 2013 – 30 June 2014).

### Evaluation design

This evaluation involved an uncontrolled pre-test post-test design. Data was collected between December 2013 and July 2014.

### Participants

The tailored MHFA course was advertised to the nursing and medical students through a variety of methods, including university course coordinators and lecturers, student clubs, student and professional peak bodies, social media and word of mouth. Participants self-selected into course delivery mode. Face-to-face course participants were recruited to this study by a research assistant or through the MHFA instructor. Using a convenience cluster sampling method in metropolitan and regional Victoria, participants were approached by a research assistant before and after their MHFA course. When the research assistant was unable to attend the course to collect surveys, the participants were invited to attend through an email sent to them by the instructor on behalf of the researchers. The online participants were invited to participate via email on enrolment in the online course.

There were 434 nursing and medical students who completed both the pre- and post-course questionnaires (see Table 1 for the breakdown the number of participants in each course type). The students were completing both undergraduate and postgraduate courses and were at all stages of their course (i.e. first year to final year). The on-line participants were from universities across the country and the face-to-face participants were from universities across Victoria. A total of 66 males (15.2%) and 368 females (84.8%) participated in the research – 25 male (8.6%) and 267 female (91.4%) nursing students, and 41 male (28.9%) and 101 female (71.1%) medical students. The average age of the students was 29.2 (SD 10.59) with a range of 17–65. The average age of the nursing and medical students was 31.7 (11.50 SD) (with 40% being under 25) and 23.9 (5.60 SD) (with 99% being under 25), respectively. The percentage of nursing students who had participated in previous MHFA and other mental health training was 2.4% and 28.4%, respectively. The percentage of medical students who had participated in previous MHFA and other mental health training was 2.8% and 21.4%, respectively.

**Table 1:** **Number of participants in course type**

**Frontline group**	**Online MHFA**	**Face-to-face MHFA**	**Total**
Nursing students	171	121	292
Medical students	102	40	142
**Total**	273	261	434

### Ethics

This research was approved by the University of Melbourne Ethics Committee. Written informed consent was obtained from all participants by ticking a ‘yes’ box at the beginning of the questionnaire.

### Measures

The participants completed a questionnaire prior to commencing their MHFA course. The questionnaire covered the following information:

1. Demographics
2. Recognition of depression
3. Mental health first aid intentions
4. Mental health literacy
5. Stigmatising attitudes

#### Recognition of depression in a vignette

The survey was based on a vignette of a person with depression that was written to satisfy the *Diagnostic and Statistical Manual’s* and the *International Classification of Diseases’* diagnostic criteria for depression (Reavley and Jorm 2011a). After being presented with the vignette, respondents were asked the open-ended question: “*What, if anything, do you think is wrong with John?*”, A ‘correct’ score was received if depression was mentioned.

#### Mental health first aid intentions and confidence

In order to assess mental health first aid intentions, participants were asked: “*Imagine John is someone you have known for a long time and care about. You want to help him. What would you do?*”. The responses were scored via the quality scoring system used by Yap and Jorm (Yap and Jorm 2012). The open-ended responses to this question were randomly intermixed and scored by a research assistant who was blinded to whether they were collected at pre- or post-course. This scoring system is based on the ALGEE action plan taught in the third edition of the MHFA course (Kitchener et al. 2013a). Responses are awarded a point for each component of the action plan they mention (i.e. *Approach the person, Assess and Assist with any crisis, Listen non-judgmentally, Give support and information, Encourage appropriate professional help* and *Encourage other supports*) and an additional point per category where specific details are given (e.g. “Encourage the person to see a psychologist” would receive two points for *Encourage appropriate professional help*). Responses can receive a minimum of 0 and a maximum of 2 points per category, giving a total score representing the quality of the response that ranges from 0 to 12.

To assess confidence, participants were asked: “*How confident would you be in your ability to help John?*” and responded on a 4-point Likert scale from ‘very confident’ to ‘not confident at all’.

#### Mental health knowledge

The students also answered 20 true or false questions based on the content of the MHFA course, e.g. “A person with a psychotic illness is less likely to relapse if they have a good relationship with their family” and “It is not a good idea to ask someone if they are feeling suicidal in case you put the idea into their head.”

#### Stigmatising attitudes

Stigmatising attitudes were assessed with two sets of statements, one assessing the respondent’s personal attitudes towards the person described in the vignette (personal stigma) and the other assessing the respondent’s beliefs about other people’s attitudes towards the person in the vignette (perceived stigma). The items were adapted to be suitable for young people (Jorm et al. 2005) based on a scale for adults (Griffiths et al. 2004; Griffiths et al. 2006). The personal stigma items were: (1) People with a problem like John’s could snap out of it if they wanted; (2) A problem like John’s is a sign of personal weakness; (3) John’s problem is not a real medical illness; (4) People with a problem like John’s are dangerous; (5) It is best to avoid people with a problem like John’s so that you don’t develop this problem; (6) People with a problem like John’s are unpredictable; and (7) If I had a problem like John’s I would not tell anyone.

The perceived stigma items covered the same statements but started with “Most other people believe that…” Ratings of each were made on a 5-point Likert scale ranging from ‘strongly agree’ to ‘strongly disagree’. Previous analyses have indicated that these items can be combined into the following scales: ‘Personal weak not sick’, ‘Personal dangerous/unpredictable’, ‘Perceived weak not sick’ and Perceived dangerous/unpredictable’ (Yap et al. 2014). Higher scores indicate more stigmatising attitudes.

Self-reported willingness to have contact with the person described in the vignette was measured by a social distance scale suitable for young people (Jorm et al. 2005) which was an adaptation of a scale developed by Link et al. for adults (Link et al. 1999). The items rated the person’s willingness to (1) go out with John on the weekend; (2) to invite John around to your house; (3) to go to John’s house; (4) working closely with John on a project; (5) to develop a close friendship with John. Each item was rated on a 4-point scale ranging from definitely willing to definitely unwilling.

### Post-course survey

The post-course survey questionnaire replicated the pre-course survey with two exceptions: the demographic questions were excluded and questions about satisfaction and quality of the course were included. Participants rated the course using a 5-point Likert scales, rating how much they enjoyed the course, how well they thought the course was structured, and how much they liked the various aspects of the course (e.g. written information, videos, activities).

### Statistical analysis

The McNemar test was used for analyzing change in the ability to recognize depression. Paired sample t-tests were used to analyse change in the mental health first aid intentions, mental health knowledge, desire for social distance, and personal and perceived stigma scores. Cohen’s d was used to measure effect sizes of changes from pre- to post-couse. Analyses were carried out using Statistical Package for Social Sciences (SPSS v22).

## Results

### Recognition of depression in the vignette

The percentages of participants who were able to recognise depression in the vignette are shown in Table 2. Recognition was very high at both pre- and post-test. The only significant change was for nursing students doing the face-to-face course.

**Table 2:** **Changes in the recognition of depression**

	**Pre-course**	**Post-course**	**P value**
Nursing students			
Online	92.4%	94.7%	.45*
Face-to-face	90.1%	95.9%	.04*
Medical students			
Online	99.0%	98.0%	1.00*
Face-to-face	92.5%	100%	.25

Note: McNemar Chi Square test; *Binomial distribution used.

### Mental health first aid intentions, mental health literacy and stigma

There were statistically significant changes in the online and face-to-face nursing students scores for mental health first aid intentions, confidence, mental health knowledge, desire for social distance, and ‘Personal weak not sick’ and ‘Personal dangerous/unpredictable’ stigma scores. There was also a significant change in the face-to-face nursing students on the ‘Perceived dangerous/unpredictable’ stigma score.

For the online and face-to-face medical students, there were statistically significant changes in the mental health first aid intentions, confidence, mental health knowledge and personal stigma scores. There were also significant changes in the desire for social distance scores in the online medical student group (see Tables 3 and 4 for pre- and post-course scores).

**Table 3:** **Changes in nursing students’ mental health first aid intentions, confidence, knowledge and stigma**

	**Online**				**Face-to-face**			
	**Pre-course Mean (SD)**	**Post-course Mean (SD)**	**P value**	**Cohen’s d**	**Pre-course Mean (SD)**	**Post-course Mean (SD)**	**P value**	**Cohen’s d**
**MHFA intentions**	3.69 (1.74)	5.47 (2.79)	.01	0.77	2.88 (1.34)	5.51 (2.30)	.00	1.40
**Confidence**	2.35 (.75)	3.30 (.56)	.00	1.44	2.39 (.73)	3.26 (.59)	.00	1.31
**Knowledge**	13.5 (2.27)	16.0 (2.30)	.00	1.09	13.2 (2.17)	15.5 (2.17)	.00	1.06
**Personal stigma – Weak not sick**	1.66 (.57)	1.53 (.60)	.00	0.22	1.75 (.64)	1.56 (.59)	.00	0.31
**Personal stigma – Dangerous and unpredictable**	2.12 (.59)	1.95 (.70)	.01	0.26	2.20 (.62)	1.85 (.75)	.00	0.51
**Perceived stigma – Weak not sick**	3.76 (.60)	3.72 (.64)	.46	0.06	3.61 (.67)	3.71 (.78)	.06	0.14
**Perceived stigma – Dangerous and unpredictable**	3.56 (.72)	3.64 (.70)	.14	0.11	3.50 (.69)	3.71 (.78)	.00	0.29
**Social distance**	2.00 (.67)	1.84 (.65)	.00	0.24	2.10 (.71)	1.90 (.69)	.00	0.29

Note: Paired sample t test.

**Table 4:** **Changes in medical students’ mental health first aid intentions, confidence, knowledge and stigma**

	**Online**				**Face-to-face**			
	**Pre-course Mean (SD)**	**Post-course Mean (SD)**	**P value**	**Cohen’s d**	**Pre-course Mean (SD)**	**Post-course Mean (SD)**	**P value**	**Cohen’s d**
**MHFA intentions**	4.02 (1.71)	6.71 (2.96)	.00	1.11	3.83 (1.84)	6.08 (2.77)	.00	0.96
**Confidence**	2.11 (.63)	3.17 (.51)	.00	1.85	2.95 (.81)	3.33 (.58)	.00	0.54
**Knowledge**	4.02 (1.71)	6.71 (3.00)	.00	1.10	3.83 (1.84)	6.08 (2.77)	.00	0.96
**Personal stigma – Weak not sick**	1.54 (.44)	1.38 (.42)	.00	0.37	1.64 (.46)	1.49 (.39)	.01	0.35
**Personal stigma – Dangerous and unpredictable**	2.14 (.55)	1.85 (.54)	.00	0.53	2.20 (.63)	1.88 (.69)	.00	0.48
**Perceived stigma – Weak not sick**	3.57 (.69)	3.60 (.68)	.60	0.04	3.58 (.56)	3.62 (.74)	.67	0.06
**Perceived stigma – Dangerous and unpredictable**	3.42 (.68)	3.42 (.78)	.92	0.00	3.46 (.69)	3.53 (.84)	.53	0.09
**Social distance**	2.11 (.67)	1.93 (.58)	.00	0.29	2.11 (.62)	2.01 (.59)	.19	0.17

Note: Paired sample t test.

### Student satisfaction

**Figure 1:**
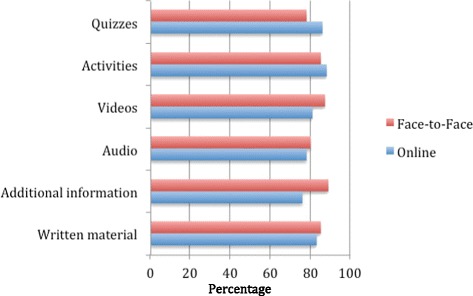
Percentage of people who rated the various aspects of the online and face-to-face training as good or very good.

Overall, the majority of participants rated the course positively, with 85% of the online participants and 88% of the face-to-face participants stating they enjoyed the course. Ninety-one percent of both the online and face-to-face participants rated the course as well structured, and 92% of the online participants and 96% of the face-to-face participants rated the course as well structured. Figure 1 presents the data rating the various aspects of the training.

## Discussion

The results of the study show that both the online and the face-to-face MHFA tailored courses for medical and nursing students are beneficial. Both types of training improved the quality of first aid intentions towards a person experiencing depression, and increased mental health literacy and confidence in providing help to someone who is experiencing depression. The training also decreased stigmatizing attitudes and desire for social distance from a person with depression. Research indicates that decreasing stigmatising attitudes and increasing mental health knowledge has the potential to increase appropriate, and decrease inappropriate first aid behaviours (Jorm et al. 2005; Rossetto et al. 2014; Yap et al. 2012; Yap and Jorm 2011).

The finding that the ability to recognize symptoms of depression did not improve significantly from pre- to post-course is likely to be explained by ‘ceiling effects’ as the percentages of people able to recognise depression before training was 92%. The pre-course recognition scores in this study were higher than in a national survey of adults and youth (Reavley and Jorm 2011b) and in a survey of Australian university students (Reavley et al. 2012b). This may be attributed to public health campaigns about recognising and getting treatment for depression, including MHFA, *beyondblue* and the Black Dog Institute (Dumesnil and Verger 2009) or may be a result of the high number of participants (68.7%) who had previous mental health training, including mental health subjects that are a part of their nursing or medical course.

It is unclear why perceived stigma scores did not change significantly for both groups of medical students and the online nursing students, however this finding is consistent with other MHFA course evaluation studies (Jorm et al. 2010a; Jorm et al. 2010b). This finding is likely explained by the purposes of the MHFA course - the goal of MHFA training is to change participant’s attitudes, not to change how they perceive others’ attitudes. It is also unclear why the social distance scores for the face-to-face medical students did not significantly change, however this may be due to lack of statistical power given the small size of this group (n = 40).

The findings indicate that the online course and the face-to-face course are similarly effective in providing MHFA training, although the comparison is limited because the delivery mode was not randomised. These findings are in line with current research comparing the effectiveness of online and face-to-face delivery methods (Means et al. 2009; Sitzmann et al. 2006). A previous study compared a CD-ROM version of the first edition standard MHFA course with a wait-list control in a randomised controlled trial and found that it increased aspects of knowledge, reduced stigma, increased confidence and improved first aid actions taken (Jorm et al. 2010b). Another potential delivery method option is a blended mode, which involves both on-line and face-to-face components. Blended delivery has been found to be preferable to face-to-face delivery (Means et al. 2009) and online learning (Sitzmann et al. 2006), but blended mode has yet to be evaluated with MHFA training.

The major limitation of this research was the lack of a control group. However, a meta-analysis of MHFA trials found that uncontrolled trials produced similar effect sizes to controlled trials, suggesting that uncontrolled trials such as the current study produce an unbiased estimate of the effects (Hadlaczky et al. 2014). Another limitation is the lack of a follow-up measure of behavioural changes as a result of MHFA training. However, in a large community sample, M Yap and A Jorm (2012) found that young people’s mental health first aid intentions predicted first aid actions taken to help a loved one with mental health problems two years after their MHFA course. Given our finding of improved intentions, we might expect similar subsequent behaviour changes in the current group of students.

Another limitation was that our sample did not match national norms with regards to age and gender. Our nursing students were slightly older (average of 32 years and 40% being under 25) than a study of nursing students (Gaynor et al. 2007) at 10 universities in 2 Australian states (51% under 25). Females were also slightly over-represented in our nursing sample, with 9% of participants being male versus 14% of the previously cited Australian study sample. The medical students who participated in this study were slightly younger than national medical student norms (Project Team 2012), with 99% of our participants under 25 versus 81% of the national medical student population being under 25. Our gender ratio for medical students was 71% female and 29% male, while nationally the male to female ratio for medical students is almost even (49% females and 51% males). One final limitation worth mentioning is that we were unable to control the timing of when participants completed the pre- and post-course surveys, particularly in the online participants. This means that participants completed the surveys at different intervals before and after completing the course.

This research contributes to the current literature on the value of MHFA training, demonstrating that both modes of delivery are effective. It also lays the groundwork for future research including comparing the efficacy of online, face-to-face and blended course delivery utilising randomisation. Furthermore, a follow-up study investigating MHFA behaviours in nursing and medical students who participate in MHFA training would strengthen the current findings.

## Conclusions

The results reported here support the effectiveness of both face-to-face and online MHFA course delivery. Both delivery methods improved mental health literacy and mental health first aid skills, and reduced stigma in nursing and medical students. This course has the potential to improve outcomes for students with mental health problems, and may benefit the students in their future professional careers.
